# Challenges and Priorities of Municipal Solid Waste Management in Cambodia

**DOI:** 10.3390/ijerph19148458

**Published:** 2022-07-11

**Authors:** Dek Vimean Pheakdey, Nguyen Van Quan, Tran Dang Khanh, Tran Dang Xuan

**Affiliations:** 1 Graduate School of Advanced Science and Engineering, Hiroshima University, 1-5-1 Kagamiyama, Higashi-Hiroshima 739-8529, Japan; v.pheakdey@gmail.com (D.V.P.); nvquan@hiroshima-u.ac.jp (N.V.Q.); 2 Department of Hazardous Substance Management, Ministry of Environment, Phnom Penh 120101, Cambodia; 3 Agricultural Genetics Institute, Pham Van Dong Street, Hanoi 122000, Vietnam; tdkhanh@vaas.vn or; 4 Center for Agricultural Innovation, Vietnam National University of Agriculture, Hanoi 131000, Vietnam; 5 Center for the Planetary Health and Innovation Science (PHIS), The IDEC Institute, Hiroshima University, 1-5-1 Kagamiyama, Higashi-Hiroshima 739-8529, Japan

**Keywords:** composting, incineration, landfill, municipal solid waste, recycling, waste treatment

## Abstract

Municipal solid waste (MSW) management is one of the utmost challenges for Cambodia’s city and district centers. The unsound management of MSW has detrimentally affected the environment and human health. In the present study, an attempt has been made to provide a comprehensive insight into the generation and characteristics, policies and legislation frameworks, management arrangement, collection, treatment, and disposal of MSW. The experience of developed and developing countries and the challenges and priorities of MSW management in Cambodia are also highlighted. In Cambodia, about 4.78 million tons of MSW were generated in 2020, with a 0.78 kg/capita/day generation rate. Only 86% of cities and districts have access to MSW collection services. The current practice of MSW management is reliance on landfill (44%). There are 164 landfills operating countrywide, receiving about 5749 tons of MSW per day. Recycling, incineration, and composting share 4%, 4%, and 2% of MSW generation, respectively. In 2021, the total revenue that was recovered from recyclables was USD 56M. The study concludes several major challenges and proposes valuable suggestions, which may be beneficial for the improvement of the current system to support the sustainable management of MSW in Cambodia.

## 1. Introduction

Solid waste management is an emerging concern for countries around the world, particularly developing nations with limited financial resources, lack of technologies, and an absence of policy framework [1,2,3,4]. In 2016, global waste generation was estimated at 2.01 billion tons with per capita generation at 0.74 kg/day, and the East Asia and Pacific countries shared the highest proportion (23%), followed by Europe and Central Asia (20%) and South Asia (17%) [5]. Developed countries seem to have a higher annual growth rate at 3.2–4.5%, while the yearly growth rate in the developing world is 2–3% [6]. An increase in waste generation is generally associated with population growth, rapid urbanization, and national GNP [1].

Rapid economic growth in recent years has spurred Cambodia to devote effort to waste management challenges [7]. Solid waste in Cambodia is classified into three categories: household waste, commercial waste, and industrial and hazardous wastes (including medical waste) [8,9]. However, there is no consensus on this classification, and the up-to-date data are relatively limited [10]. Municipal solid waste (MSW) combines wastes from households and commercials [11], generated from households, markets, restaurants, shops, hotels, offices, street sweepings, and miscellaneous [12,13]. Rapid urbanization and industrialization have led people to migrate to cities to obtain better jobs, a higher education, and access to health care services, making waste generation unpredictable. The data on MSW generation are mainly estimated based on population and constant generation rate in the entire country, and the reliable data of MSW generation are hard to come by. 

MSW composition is primarily food and organic waste (52–63%) [12,13], usually disposed of by mixing with plastic, glass, textile, paper, and other fractions without separation at the sources. An increase in population, rapid urbanization, economic growth, and improved living standards are the main factors affecting MSW generation, in terms of both quantity and quality [14]. The change of lifestyle and consumption patterns is also a driving force behind a rapid increase in MSW in the country [15]. For example, the use of plastic cups, plastic straws, and plastic bags for takeaway coffee is becoming more popular in Cambodian society. Another example is using plastic bags and Styrofoam containers for packaging food instead of reusable containers. These practices may not only pollute the environment but also negatively affect human health. Jadhav et al. illustrated that using plastic materials for packaging food and beverages releases microplastics and toxic substances, which may be harmful to consumer health [16].

A rapid increase in MSW in urban areas has put pressure on the collection and management system. The collection service is mostly prioritized in populated areas with good infrastructure and high commercial activities due to lack of transportation and low revenue collection [13,17]. In 2021, approximately 2.10 million tons of MSW were collected from 204 city and district centers [12] with an average rate of 72% [18]. The collection rate varies by city and district. Phnom Penh, the municipality of Cambodia, has the highest collection rate at 91–92% [5,19]. The limitation of public awareness and participation also poses a challenge for the environmentally sound management of MSW. Cambodia has no large-scale waste treatment and recycling facilities. The lack of waste treatment technologies and infrastructures has shortened the lifespan of landfills since most MSW collections are directly sent to landfills without intermediate treatment [17]. The effort to reduce and reuse refuse materials is still weak, and the management of landfills is poor. In recent years, the government has made many efforts to enhance MSW management by developing new regulations, policies, strategies, and guidelines. However, the question is how such instruments can be effectively implemented towards the sustainable management of MSW by taking account of environmental, economic, and social aspects.

This paper aims to identify the challenges and opportunities of the current MSW management in city and district centers of Cambodia, of which the major objectives are: (1) to review the status of MSW management systems, focusing on the technical, legal framework, and institutional arrangement; (2) to share experience from other developed and developing countries in management, treatment, and supporting policies; (3) to identify key priorities to improve the status and effective use of resources from waste.

## 2. Methodologies and Search Strategy

This paper presents the current practice of MSW management in urban areas of Cambodia. The study is based on the available data, governmental-related regulations and policies, reports, and published papers. The data describing the MSW generation (2008–2020), collection, recycling, landfilling, and incineration were obtained from the Department of Solid Waste Management of the Ministry of Environment (MoE). The legal instruments, policies, and strategic plan were collected from the MoE and its official website, while the management arrangement and treatment technologies were reviewed based on published articles and reports of the MoE. The reputable references were searched from the ScienceDirect, PubMed, and Google Scholar databases by using the key terms containing MSW management system, MSW collection and transportation, waste treatment technology, recycling and disposal, and challenges in MSW management in Cambodia and selected developed and developing countries. Only English and Khmer documents were selected as references for this review work.

## 3. MSW Generation, Characteristics and Composition

### 3.1. MSW Generation

MSW has been observed to increase substantially in the past decades and vary from place to place. The generation of MSW is generally associated with population growth, economic conditions, standard of living, and urbanization [13,20,21,22,23]. However, the robustness of national data in Cambodia is hard to obtain and inconsistent among the sources, except for Phnom Penh Municipality (PPM), where the data are more available and reliable after installing the weighting bridge at the landfill site [13]. 

The aggregate data of MSW throughout the country revealed a linear increase trend from 2.50 million tons in 1990 to 4.24 million tons in 2016 [24]. Akenji et al. reported 4.09 million tons of MSW generation with a generation rate of 0.73 kg/capita/day in 2015 [9]. The MoE estimated that approximately 4.78 million tons of MSW were generated in 2020 with per capita generation at 0.78 kg/day, as shown in Table 1, while the global average per capita was 0.74 kg/day [5]. An increase in MSW in the cities and districts of Cambodia is significantly influenced by population growth and the movement of people from rural to urban areas [10]. In addition, the World Bank reported that a rapid increase in MSW in Cambodia was caused by an improvement of living standards and an increase in economic activities from the tourism, construction, and industrial sectors [25].

Urbanization has positively influenced MSW generation in developing countries where the gap in economic activities and living standards between rural and urban areas is significant, and MSW per capita in the urban environment has shown to be higher than that in rural areas [28]. Furthermore, the difference is also observed among cities; high-income cities are more likely to generate more MSW per capita than those with lower incomes [1]. For example, a recent rapid development, per capita MSW generation in PPM was 1.32 kg/day in 2016 [29], almost double the country’s average value [9,27]. 

The global MSW generation is expected to increase from 2.01 billion tons in 2016 to 3.40 billion tons by 2050, and the low- and middle-income countries are projected to have the highest increase rate, by at least 40% [5]. In Cambodia, MSW generation is expected to increase by 36% by 2050 with per capita generation from 1.16 kg/day in 2030 to 2.13 kg/day in 2050 [24]. This drastically increasing trend may cause landfills, particularly in large cities such as Phnom Penh, Sihanouk Ville, Siem Reap, and Battambang, to rapidly reach their capacity limit. 

### 3.2. MSW Characteristics and Composition

Waste characteristics greatly depend on socio-economic conditions [30]. Other factors such as food habits, cultural traditions, climate, and socio-economic factors of households, including average family size, house size, employment status, and income, significantly influence waste characteristics and composition [11,31]. MSW typically includes food waste (kitchen waste, food waste, vegetables, snacks, etc.); paper (office paper, newspaper, booklets, cardboard, etc.); plastic (plastic bottles, PET bottles, plastic bags, foam plastic, etc.); metals (ferrous can, ferrous scrap, aluminum scrap, copper, etc.); textile (cloth, fabric, cotton, etc.); glass (glass bottles, broken mirrors, etc.); wood and dry matter (branches, grass, trim, etc.); and others (ceramic, brick, inert materials, stone, etc.). Bulky wastes such as furniture and packaging materials are broken up into pieces before disposal. Food waste is the most dominant garbage in all locations (Table 2), in which Kampong Chhnang has the highest proportion, while the lowest is Phnom Penh. Plastic waste was the second top, followed by paper.

**Figure 1:**
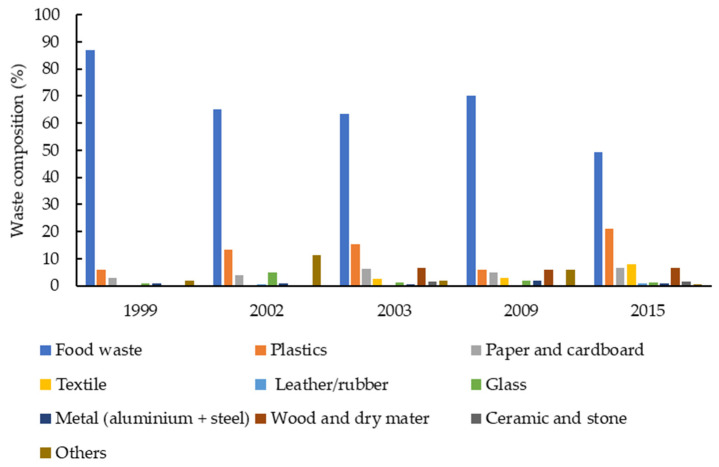
Waste composition in Phnom Penh municipality. Adapted from [12,33,34,38].

In PPM, the composition of MSW has changed over the years. Food waste drastically declined from 87% in 1999 to less than 50% in 2015, while plastic waste showed an increasing rate from 6% to 21% in the respective years (Figure 1). Changes in waste composition are mainly due to the economic boom and changes in living standards and consumption behaviors [15]. The information on waste composition is significant for selecting and developing appropriate handling methods and treatment technologies based on each type of waste [37].

## 4. Legislation and Policy Framework

Striving to improve the MSW management system, numerous regulations, policies, and guidelines have been developed and adopted. Under the Law on Natural Resource Management and Environmental Protection (1996), the MoE is responsible for developing regulations, guidelines, and monitoring waste management, including hazardous, industrial, and medical wastes [39]. In 1999, the sub-decree No. 36 on Solid Waste Management came into force [40]. Under this sub-decree, solid waste is classified as garbage, solid waste, and hazardous waste. The municipality and city authorities are responsible for collecting, transporting, storing, recycling, reducing, and disposing of garbage and hazardous waste. The importation of all types of waste from other countries is strictly prohibited.

A more comprehensive sub-decree, No. 113 on Municipal Solid Waste Management, was developed in 2015, and the responsibility of overall MSW management was handed over to municipality and district authorities [17,41]. The transformation of management function allows the municipality, city, and district authorities to deliver the waste collection service or contract the service to a private operator. The sub-decree also sets the roles of waste generators in sorting out recyclable waste and packing separately. However, the sub-decree does not regulate the standard of bins and plastic bags. This regulation has also set the measurement of landfill management, but it does not define the technical standard of leachate and gas control systems. In 2015, the MoE set the standard level of 84 chemical substances in soil and another 74 chemical substances that are allowed to be disposed of at the designated landfills [42].

To support the city authorities in the implementation of sub-decree No. 113, the government allocated USD 5M of the national budget for MSW management in the 26 piloting cities, except PPM, based on the inter-ministerial declaration No. 73 on the Usage of Environmental Sanitation Service Fund in Cities (2015) [43]. The amount of budget distribution to each city is associated with the population size and subjected to an increase every year. Furthermore, each city is empowered to make decisions on the recruitment of private operators and using funds for waste collection services. However, only 17% of the cities integrated MSW management into their 5-year development plan and controlled the service that was provided by private operators [44]. The reason behind this was that the cities were not given full authorization of the management function of MSW service and lacked guidance from the national level prior to taking action [44].

In 2016, the MoE issued a declaration on the management of battery waste [45]. All types of battery waste must be kept separately from other waste, avoiding any leakage of hazardous substances into the environment. Nevertheless, implementation on the ground has been slow and challenging due to the lack of mechanisms for collecting, storing, and treating disposal battery waste. In 2021, the MoE piloted battery waste collection by installing 74 battery waste bins at 68 locations in PPM and 11 other provinces [18]. Another sub-decree, No. 16 on Electrical and Electronic Equipment Waste Management, was endorsed in 2016 to prevent any illegal disposal of these e-wastes. Sub-decree No. 168 on the Management of Plastic Bags was adopted in 2017 to promote biodegradable plastic and public participation in plastic bag reduction through the reuse of plastic bags or the utilization of eco-friendly bags [46]. The sub-decree is specifically restricted to the importation, production, distribution, and use of plastic bags. Only plastic bags that meet a minimum threshold of 0.03 mm with a base width that is larger than 25 cm are permitted to be imported and produced locally. Supermarkets and shopping centers are prescribed to charge customers USD 0.10 per bag. Although this rule has been in force since 2018, its implementation has not yet been fully implemented. A study of UNDP with six supermarkets indicated that only four of them effectively implemented the fee charge from customers, while the others gave the bags away for free [47]. Like in other countries, the circular economy has been adopted in Cambodia by shifting a traditional linear economy model to a more environmentally friendly model. Instead of mass production and mass disposal, waste is used as a secondary resource to replace raw materials for production [9]. Hence, in 2019, the MoE established a plastic task force to promote the 4Rs (refuse, reduce, reuse, and recycle) mechanism to reduce plastic waste. The task force focuses on three main areas consisting of: (1) reviewing plastic-related policies and regulations; (2) outreach and communication regarding plastic waste-related topics; (3) developing and supporting plastic-related businesses.

Many reforms are underway in MSW management in Cambodia. In addition to the above regulations, the government has released several policy instruments. The latest is the Municipal Solid Waste Management 2020–2030 Policy, which targets the initiation and implementation of a new advanced management system by considering economic efficiency, financial resource capacity, environmental sustainability, and social acceptance. The policy is designed to support the government Rectangular Strategy Phase IV in ensuring environmental sustainability and readiness for climate change by prioritizing strengthening the management of solid, liquid, gas, and hazardous wastes through the implementation of the 4Rs. In this sense, waste is regarded as a resource, and waste generators are obligated to pay the management fee.

## 5. Institutional Arrangement

**Figure 2:**
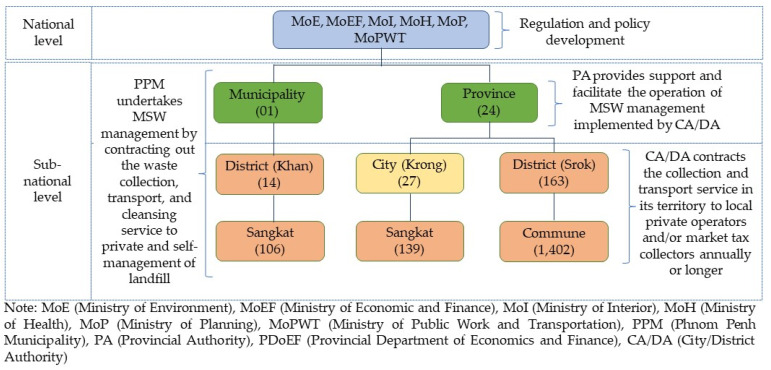
MSW management arrangement in Cambodia.

MSW collection in some main cities has recently been improved through the delegation of functions from the national level to the sub-national level. Greater endeavor is required to improve the SMW and hazardous waste, e-waste, and plastic waste in all municipalities and cities. There are two levels of MSW management in Cambodia: (1) national level (line ministries); (2) sub-national level (provincial, municipality, city, and district authorities), as shown in Figure 2. The national level management mainly focuses on policy design, legislation development, capacity building, monitoring, and supervision. Meanwhile, the sub-national authorities are responsible for implementing the policies and the operation of the MSW management system directly or through the private sector. At the national level, the MoE is mandated to develop related waste management regulations and guidelines, monitors the executive of sub-national levels, enforces related law, and issues disposal permit for industrial waste. The Ministry of Health (MoH), Ministry of Planning (MoP), and Ministry of Economic and Finance (MoEF) are involved in medical waste management, development planning, and approval of the sector financial investment, respectively. The Ministry of Interior (MoI) engages with strengthening the capacity of sub-national functions and managing the Environmental Sanitation Service Fund, while the Ministry of Public Work and Transportation (MoPWT) oversees the landfill design and construction. Despite this, financial, technical, and human sources are the main challenging issues in respect of effective MSW management, which requires institutional reforms, new strategy development, and the improvement of the collection system [48]. In 2021, the National Committee for Municipal Solid Waste Management was established, consisting of members from relevant ministries and institutions and chaired by the environmental minister. The committee is in charge of developing waste-to-energy (WTE) related policies, strategies, action plans, and monitoring mechanisms [49]. The committee also aims to play a vital role in promoting environmentally friendly products and raising citizens’ awareness. 

At the sub-national level, the municipality, city, and district authorities are responsible for the collection, transportation, storage, recycling, and dumping of MSW with support and coordination from provincial authorities [41,48]. Management capacity, personnel, and financial allocation have limited the capacity of sub-national authorities to handle the service. As a result, the MSW management contract was awarded to private operators in many cities and districts. In PPM, the management of MSW is centralized at municipal administration, and it is mature compared to other cities and districts. The Phnom Penh Waste Management Authority (PPWM) has been responsible for MSW management by franchising the collection, transportation, and cleansing service to a private company (CENTRI) for 49 years. CENTRI has a monopoly on the MSW collection service within the municipality and has owned the sole rights to collect service fees to cover its operational and disposal costs since 2002 [13]. However, the service that is provided by the company had not been improved, and many complaints have been made by residents [17]. In 2019, the government decided to reform the waste collection service in PPM and divide the city into three operational zones. One year later, the government terminated the contract with CENTRI and put service rights up for bidding. In this regard, three companies were recruited and operated in three different zoning areas in mid-2021. In addition, the service contract has shifted from a conventional non-monetary compensation contract (service serves based on revenue collection from generators to cover the operational cost) to a fee-based contract (PPM pays the service fee at a fixed rate to private operators). In this sense, the PPM administration takes on the role of service fee collection.

Although the management of MSW has been decentralized to cities and districts, the authorities are not clear about their responsibility but still fully implement their roles [48]. Based on sub-decree No. 113, city and district authorities are assigned the responsibility of waste management through the delegate function mechanism, while provincial authorities are responsible for the overseas implementation of cities and districts concerning MSW management. In some cities and districts, the role of provincial authority in MSW management to some extent overlaps with city and district authorities. For instance, in Kep province, provincial authorities undertake waste management and contract out the service to private operators, which overlaps the roles of city and district authorities that are set out in sub-decree No. 113 [7]. In most cases, cities and districts face difficulty finding private operators due to small service coverage, the low willingness of people to use the service, long distance to the landfill sites, and no economic viability. Therefore, the MSW collection service is somehow outsourced to the market tax collector, community, and voluntary group and limited to a small coverage area.

## 6. MSW Management and Treatment

**Figure 3:**
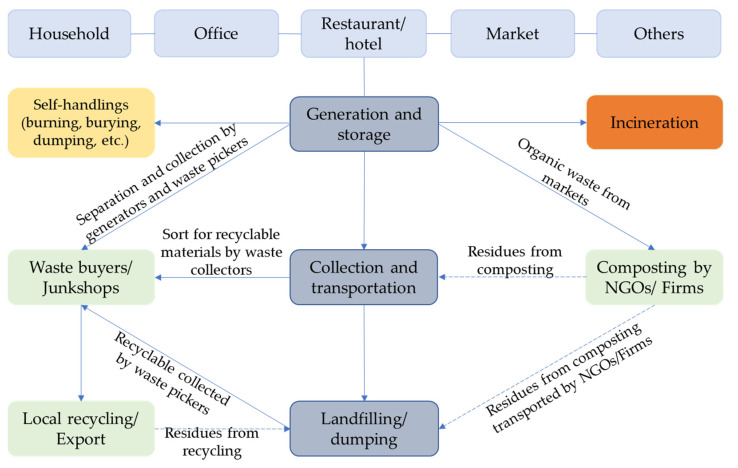
Diagram of MSW management stream in Cambodia.

MSW management refers to the process of managing waste in a particular area, and the treatment is mainly focused on facilities that convert waste into resources before final disposal. Municipalities, cities, and districts have developed their infrastructure quickly to meet the latest developments and become more urbanized. The waste collection service coverage in many cities is also expanding. However, the services are appropriately managed only in the central cities of Phnom Penh, Preah Sihanouk, Siem Reap, and Battambang [8,50]. The current MSW management stream is characterized into three phases: generation and storage at the sources, collection and transportation by private operators and local authorities, and final disposal at the landfill sites (Figure 3). The industrial waste is collected separately from the MSW and transported to industrial waste disposal sites by licensed operators. However, industrial waste is sometimes illegally collected by mixing it with MSW due to an irregular collection schedule.

### 6.1. Generation and Storage

The existing legal instruments do not set the standard for waste bins and package bags. MSW is generally mixed and packed in a plastic bag without separation. Residents pack their household waste in reused plastic bags without caring about the size and color and store it in the bin at the curbside or outside the gate. The MSW is customarily packed in a similar way to household waste and stored in larger bins at institutional and commercial buildings such as restaurants, hotels, and shops. The collection of waste is performed manually from door to door. This collection model is time-consuming, reducing vehicle productivity and the productivity of the workforce that is deployed [38]. Waste from the local market in central cities is disposed of at the designated public space, which is later collected by workers using a shovel; or, it is kept in the hauling containers that are provided by waste collection companies that could be mechanically loaded into collection trucks and transported to the landfills. According to [38], waste that is placed outside the house along the curb or roadside is scattered by animals searching for something to eat or scavengers searching for recyclable materials. Due to a limited storage system, Kum et al. [37] suggested that the standard bins or containers should be introduced for commercial areas, and the appropriate storage system established.

Sorting out at the generation sources is a fundamental method in separating waste, contributing to a decrease in waste volume, recovering valuable resources, lessening landfill size, and reducing costs on waste collection, transportation, and treatment [51]. The government has promoted the 3Rs (reduce, reuse, and recycle) approach since 2008 through the National Strategy on 3Rs on Waste Management. However, the strategy seems to be dysfunctional due to the absence of regulation and supporting mechanisms. The success of the 3Rs depends largely on active participation and an awareness of waste generators [52]. To encourage source segregation, in 2021, PPM issued a waste separation rule and warned of non-compliance actions. The rule demands residents to classify waste into two categories: wet and dry wastes. Wet waste, including organic and kitchen wastes, is packed in black plastic, while dry waste (paper, cardboard, plastic, metal, bottles, etc.) is placed in white plastic [53]. At the same time, hazardous waste such as glass and sharp objects are kept separately and collected every Sunday. Bulky wastes such as tree branches, construction and demolition waste, e-waste, and tire waste are required to be stored properly. Waste generators must contact and request a waste collection company to dispose of bulky waste. However, the rule does not indicate if there is an extra charge for bulky waste disposal. The rule also set a daily schedule for wet waste collection, or twice a week (Monday and Friday) for dry waste. In case of non-compliance with the rule, waste generators can face fines and waste collectors can refuse to collect. Other cities, except Sihanouk Ville, are yet to adopt this system.

### 6.2. Collection and Transportation

MSW collection, management, and disposal are the main environmental challenges for urban areas [37]. As mentioned earlier, waste collection and transportation services are the sole responsibility of local authorities. However, the authorities have transferred the service to private operators due to limited budget and personnel, and a lack of regulations, standards, and guidelines [48]. The service provision mainly refers to the profit margin calculations from waste fee collection and negotiation with national and sub-national authorities [44]. Therefore, waste management is typically based on the revenue that is collected from service users. The fee for waste collection is calculated based on the type of building and number of floors for residential waste, and the types of business, such as restaurants, guesthouses, clinics, schools, etc. [48]. There was no standardization on the service fee calculation, and some complaints were raised regarding an excess of tariff that was set by authorities [48]. The investment and operational costs are financially different from city to city. In the MSW management system, costs on collection and transportation share the highest proportion at 82% [1]. Due to this high operational cost, private operators generally receive subsidies or donations from the government or development partners, or through a public–private partnership scheme [17]. According to Kaza et al., about 50% of the investment cost is covered by the local government, while the remaining portion is contributed by the government, private operators, and other funding sources [5]. 

The service that is provided by private operators is somehow inefficient and does not comply with the terms of reference set out in the agreement [54]. The reason behind this backdrop is that the private operators face financial challenges due to unclear service fees, a lack of performance benchmarks in the contract, inefficient fee collection, and an unwillingness to pay the service as the private operators themselves collect the fee from waste generators [13,17]. A lack of operation monitoring and enforcement is also a driving factor for poor service provision [54]. As a result, private operators focus on the more profitable areas with good infrastructure, more institutions, economic activities, and commercial buildings, and dwindle the service in low-paid areas.

Cambodia consists of 204 cities and districts with 6.14 million people [55], generating about 2.94 million tons of MSW [18]. As illustrated in Table 3, about 86% of city and district centers implement the MSW collection service. Approximately 5749 tons (72%) of MSW generated in urban centers has been collected and sent to 701 landfills [18]. Waste collection efficiencies vary across the cities and districts. The municipality of Phnom Penh achieved a 92% collection efficiency, lower than neighboring countries such as Bangkok, the capital city of Thailand, which achieved 100% [5], and the two largest cities of Vietnam, Hanoi and Ho Chi Minh, which were 98% and 97%, respectively [5,30].

**Figure 4:**
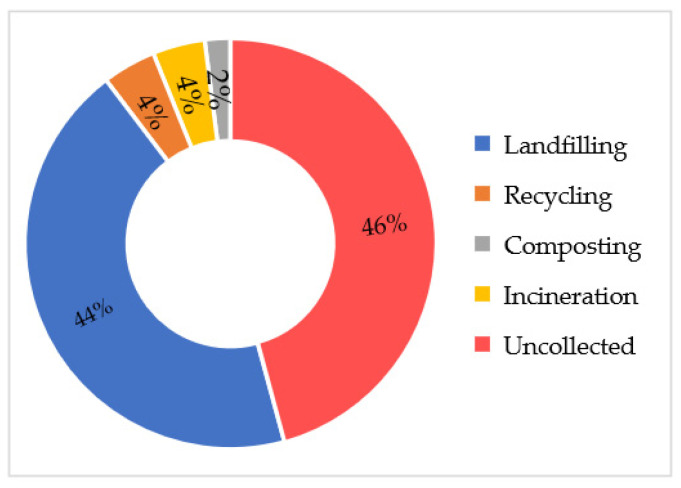
MSW treatment in the country (authors’ own calculation based on [18,24]).

Recycling, composting, incinerating, and landfilling made up about 54% of MSW in the whole country, while the remaining 46% was uncontrolled disposal (Figure 4). Out of the total MSW that was collected, 44% was sent to landfill and the rest recycled, composted, and incinerated. It is can be clearly seen that MSW collection improved from 38% in 2016 [24] to 54% in 2021. This collection efficiency is higher than that found in low-income countries (less than 50%) [1] and lower-middle-income countries at 51% [5].

**Figure 5:**
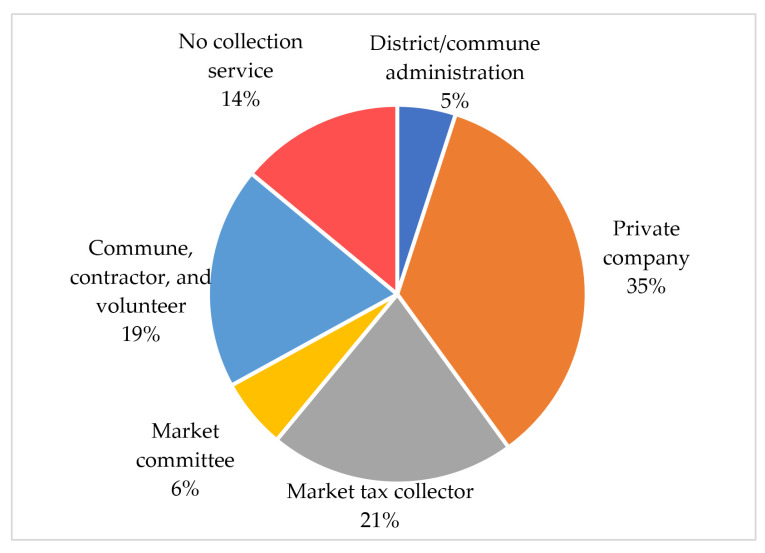
MSW collection service provision by different actors. Adapted from [27].

There are numerous types of private operators in Cambodia, such as private companies (35%); market tax collectors and market committees (27%); community groups and local contractors (19%); and local authorities (5%), as shown in Figure 5. The services that are provided by market tax collectors, market committees, and community groups are limited to waste generation from markets and surrounding residences or commune centers. The lack of transportation and personnel is the biggest obstacle to the effective management of MSW in Cambodia [54]. Moreover, Spoann et al. added that challenges in MSW management are associated with the poor conditions of waste trucks, facilities, roads, and insufficient trucks and equipment [56]. The MoE estimates that approximately USD 110M is needed to buy machinery and equipment such as compactors, arm hooking trucks, dumpsters, excavators, and bulldozers, in order to meet optimum waste collection in all cities and districts [18].

### 6.3. Landfill and Disposal

Landfill is the primary method for disposing of MSW that is collected from urban areas in Cambodia [18,57]. In 2021, there were 164 private-owned and state-owned landfills operating in the entire country, receiving about 5749 tons of MSW per day (Table 3). Landfills in the country are mainly unsanitary open dumpsites without soil cover, leachate treatment, and gas control systems. A lack of management and adequate disposal of MSW has adversely affected the environment, human health, and global warming through the emissions of GHG [37]. In some urban areas with an absence of state-owned landfills, private operators set up their own landfills. However, this landfill type is normally open dumped, without fencing and environmental pollution control measures, and commonly occupies a small land size. Burning waste at landfill sites is the only means to reduce waste landfilling and reclaim landfill space. State-owned landfills are constructed on state private land (according to the Land Law 2001, state private land refers to all property that belongs to the state but does not have a public interest value) using the state’s budget or external funding. The sub-national authorities are responsible for operating and managing these landfills and charging private operators’ gate fees. Like private-owned landfill, most state-owned landfills operate as dumpsites without soil cover, fire control system, gases collection, and leachate treatment system [13,48]. To ensure the effective management of the landfill sites, the government established a state-owned company, the Enterprise for Managing Transfer Stations and Landfills for Solid Waste (EML), in late 2020. The company is responsible for constructing and developing the transfer stations, landfills, and other treatment infrastructures, such as resource recovery facilities, recycling facilities, and WTE incineration plants throughout the country under the technical management of the MoE and MoEF [58]. This new company collects revenue from landfill gate fees, transfer stations, and landfills services to support its operation and development.

The amount of MSW sent to landfills in the whole country has increased significantly, from 317,550 tons in 2004 to 1 million tons in 2014 and 2.09 million tons in 2021 [23,27,57]. An enormous volume of MSW has been sent to the Dangkor landfill in Phnom Penh. In 2020, Dangkor landfill received 2835 tons of MSW every day [59], a triple increase compared to the amount that was accepted in 2009 [60]. The Dangkor landfill was expected to reach its capacity limit in 2021 but may possibly receive more waste from the municipality over the next few years until the new regional landfill is implemented. It is important to note that the government decided to construct a new regional engineered landfill in Kandal province, located about 25 km west of Phnom Penh. The landfill is designed for 23 years (2023–2045) on 100 ha of land. Once the new landfill is ready for operation, the Dangkor landfill will serve as a transfer station to sort out mixed waste into parts prior to recycling, incineration, and landfill. In 2021, there was only one sanitary landfill operating in Preah Sihanouk province. A dozen more are being planned and are under construction in other provinces, including Kampong Chhnang, Pursat, Battambang, Siem Reap, Banteay Meanchey, and Prey Veng.

### 6.4. Recycling

Waste recycling in the country is generally limited and relies on the informal sector [61]. As shown in Figure 3 above, the collection of recyclable waste is found in each stage of the MSW management stream. At the generation sources, some recyclable waste (mainly aluminum cans and glass bottles) is sorted by waste generators before being stored temporarily in the bin or container. However, this practice is limited, resulting in large waste disposal without source separation. Waste pickers play a crucial role in resources recovery from waste in household bins, temporary storage containers, and landfill sites. During collection, waste collection workers usually search for the remaining recyclable materials before transporting them to landfills. Recyclable materials that are recovered by generators, waste pickers, or workers are often sold to waste buyers (Edjai) or directly to junkshops. Waste buyers travel around cities with a pushing-cart to pick up and buy recycled materials. Approximately 3000 waste pickers collect a large amount of recyclable waste to sell to waste buyers and junkshops, who then export it to other countries [62]. This informal sector is often observed throughout the country, contributing to reducing MSW at landfills.

Junkshops are a critical player in buying recyclable waste from waste buyers for selling to domestic recycling enterprises or compressing materials into bulk quantities for exporting. In 2021, there were 692 junkshops operating nationwide, buying approximately 16,811 tons per month of recyclable waste (Table 4), about 5% of the total MSW generation or 9% of collected waste. Metals share the highest proportion (46.76%), followed by paper and cardboard (33.16%) and plastic (17.82%). The limit of source separation and the low cost of recycled materials have caused lower resource recovery from waste [63]. Based on the price of recyclables set by the junkshops, the total revenue of recovered materials was about USD 56M in 2021.

**Figure 6:**
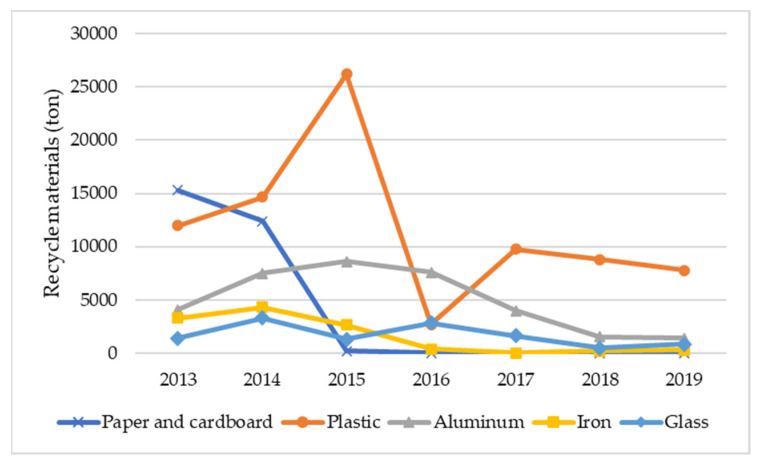
Recycled materials exported to other countries (adapted from [27]).

Domestic recycling activity also seems limited due to the lack of recycling industry and infrastructure and the market for recycled materials and products [29]. Most recyclable materials are exported to other countries with available recycling infrastructure, such as Thailand, South Korea, Vietnam, China, Singapore, Malaysia, and Taiwan [29]. However, the quantity of exported recyclables decreased substantially from 47,916 tons in 2014 to 10,387 tons in 2019 (Figure 6), as those target countries limited and banned importing waste. For example, China introduced the Green Fence Policy in 2015 by reducing the importation of low-value plastic and the National Sword Policy in 2018 by banning the importation of 24 types of waste [64]. Similarly, Vietnam halted the issuance of a plastic waste importing license in 2018 and announced the cessation of all waste imports by 2025 [65], while Thailand banned the importation of plastic waste in 2020 [62]. In this regard, there is room for new business involving waste recycling, mainly plastic recycling, since this business type is still limited in Cambodia.

The UNDP estimates that Cambodia has lost millions of dollars by exporting 300,000 tons of recyclable materials to other countries without developing its recycling sector [66]. Therefore, the government-supporting policy is required to increase materials recovery locally and minimize health and environmental risks through such recovery activities. The government should encourage recycling companies to enhance domestic recycling and provide financial subsidies to support recycling infrastructure costs [14,67]. Akenji et al. suggested that the government should encourage private sectors to develop domestic recycling infrastructures, such as the recovery of plastic waste for refuse-derived fuel (RDF), and construction materials such as roads and plastic timber [9]. Similarly, Seng et al. recommended that resource recovery and recycling markets should be the priority issue when developing a new MSW management system [33]. 

### 6.5. Composting

Composting is a practical approach to reduce waste at landfill sites [68,69] by converting biodegradable waste into fertilizer [63]. Organic waste accounts for more than 50% of the total waste in Cambodia, but value extraction from this organic material is very low. In other words, more organic waste mixed with other types of waste has been sent to landfills. Under anaerobic conditions, organic waste naturally biodegrades, generates greenhouse gases (GHGs) that affect the atmosphere, contributes to global warming [63,70,71], and contaminates water [72]. Seng et al. indicated that converting organic waste into compost and resource recovery could share about 20% of the MSW [33]. However, fewer attempts have been made in waste composting due to people undervaluing compost fertilizer, minimal landfill space for setting up composting plants, lack of waste separation, and limited technology and human resources [63]. As a result, only a few small-scale composting plants are in operation and share about 2% of MSW generation [24].

### 6.6. Open Burning

Burning waste occurs in most rural areas without waste collection services [10,24]. Waste in areas with no waste collection services is often burned, buried, and illegally disposed of on vacant land and water bodies [73]. According to Kham et al. [7], the open burning of waste is most common (57%), followed by burying (11%), dumping on vacant lands (9%), disposing of waste in water (5%), and other methods (9%). However, these handling methods have significantly decreased over the years as the waste collection service has improved, resulting in a large amount of waste being sent to landfill [24]. The lack of established waste minimization strategies and management systems are also factors affecting waste burning. Hence, reducing open burning requires a suite of interventions, including developing a 3Rs approach, waste separation and collection, the improvement of landfill management, and law enforcement.

### 6.7. Incineration

Incineration is a heating process involving the combustion of all waste materials at a high temperature to break down waste into chemical components through oxidation. The process performs at a temperature of between 750 and 1000 °C to produce heat and energy [74]. This method can reduce waste mass by 70% and waste volume by 90% [75]. Currently, there is no large-scale technology for the treatment of MSW in the country. In urban areas with limited access to waste collection services or landfill spaces, the MoE has installed small-scale incinerators without energy recovery. By 2021, 54 incinerators were installed across the country, and the burning capacity ranged between 1 and 8 tons per hour [18]. However, different compositions of MSW in Cambodia are generally mixed up without pre-processing and dominated by organic materials with high moisture content and lower heating value, which requires auxiliary fuel. Incomplete burning waste in the incinerator may produce carbon monoxide, dioxin, and other harmful substances. Therefore, incineration plants in many countries have been opposed because of their high level of toxic dioxin emissions and other pollutants. High investment costs are also required for a higher efficiency incinerator with advanced flue gas treatment [34]. Hence, the efficiency of existing incinerators in respect of air pollution emissions is still a rather open question. 

To promote the WTE sector, the government has encouraged the private sector to explore business opportunities targeting energy recovery from waste. However, the traditional WTE incinerator requires a high investment cost. A low collection rate and dispersion of waste disposal are also challenging issues for the WTE project. PPM is seen as the most promising city with a high volume of waste generation. A study of GGGI indicated that the energy output from the WTE plant is 10 MW [76]. This study suggested that the Feed-in-Tariff (FIT) should be USD 0.10 per kWh together with USD 18 per ton of gate fee. However, the present landfill gate fee in Phnom Penh is only USD 0.7 per ton, and the FIT that the electricity company buys from the solar power plant in the country is lower at USD 0.08 per kWh [77]. According to Seng et al., the WTE incineration plant is feasible for Phnom Penh. However, the high moisture content in the wet season is a challenging issue that requires advanced treatment technology [33]. Therefore, from a sustainable economic perspective, the WTE plant significantly requires subsidy and policy support from the government. A lack of supporting policy may prevent investment in WTE projects in this country. The GGGI’s study also illustrates that the conversion of MSW into RDF is another economically viable option [76]. Mechanical biological treatment (MBT) is a well-known and less expensive technology producing RDF from waste. The MBT plant sorts out metal and inert materials, recovers recyclable materials, removes moisture, and separates high calorific fraction for RDF. The potential market for RDF is the cement factory, which uses RDF to substitute coal. This co-processing technology requires a high temperature to burn toxic substances in cement kilns. It is worth noting that co-processing technology is already employed by one cement company, Chip Mong Insee Cement Cooperation in Kampot province, absorbing more than 8000 tons of industrial waste per month and contributing to the reduction in CO_2_ at 94 kt [78]. Recently, in 2021, the government established the national committee for MSW management to promote energy recovery from waste. The committee plays an essential role in developing incentive mechanisms, related policy, and legal frameworks regarding WTE projects. 

## 7. Management and Treatment of MSW in Other Countries

Table 5 illustrates the comparative management of MSW in Cambodia and that in the representatives of developed countries (USA and Japan); Asian countries (China and India); and the neighboring country (Vietnam). Accordingly, the differences in MSW management between Cambodia and other countries are analyzed based on selective parameters, which can provide a more comprehensive view of the current state of MSW management in Cambodia compared to that in the region and internationally. 

### 7.1. The United States of America

The MSW generation in the United States of America (USA) is 2.03 kg/capita/day [79], higher than that in Japan and other countries (Table 5). Like Japan, the USA has achieved 100% in the MSW collection of transportation since 2016 [1]. Source segregation is required for all residential, commercial, and institutional solid waste [79]. Waste recycling in the USA was higher than in other countries. However, a high proportion of MSW is disposed of at the landfill, which is not the environmentally favored method. In contrast, landfilling in Japan shares a minor proportion, while incineration is the primary MSW disposal method. To give a push in MSW recycling, the US government has initiated subsidy programs for recycling centers [95]. It is worth noticing that many landfill sites also receive funding from the government to improve the environmental safety of landfilling [95]. To date, 86 incinerators have been installed in the USA, consuming about 34 million tons of MSW [79]. Incineration contributed about 12.8% of MSW treatment in the USA [81].

### 7.2. Japan

In Japan, residents must separate waste following the rules that are set by local authorities, and pack the waste in designated plastic bags [96]. Waste segregation rules vary across cities, and violating these rules can result in being fined. Some cities set the classification rule into three categories (burnable, unburnable, and recyclables) [97], while some require its separation into two types (burnable and recyclables). Colored plastic bags are designated for each category. The local governments charge the service fee through selling the designated plastic bags, a standard user fee in Japan [5]. Most cities of Japan, including Osaka, Kobe, Naha, Toyama, Yokohama, and Kitakyushu, reach 100% of the waste collection rate [5]. The effectiveness of the MSW management system in Japan is a result of solid cooperation between national and local governments, as well as the participation of citizens. The Japanese government provides subsidies on infrastructure costs up to 33% and 50% for the basic and advanced high-efficiency facilities, respectively [5]. According to the Ministry of the Environment, Government of Japan, in 2019, Japan generated about 43 million tons of MSW, and only 1% was sent to landfills without intermediate treatment, while 19% was recycled, and the remainder was injected into incineration plants [98]. In 2014, Japan had approximately 1200 incinerators, of which there were 358 WTE plants, which produced electricity of 7958 GWh [99]. 

### 7.3. China

The central government of China is responsible for developing regulations and guidelines for solid waste management, while the local government takes an implementation role. The municipal governments have sole power in setting up the requirements and practices for MSW disposal [100]. An increase in domestic waste generation and a growing amount of imported waste have placed China in a waste crisis. MSW generation proliferated from 179 million tons in 2014 to 204 million tons in 2015, and it was predicted to increase to 282 million tons in 2020 [101]. Facing such a critical challenge, China issued numerous policies to ban the importation of waste in 2017, in order to achieve its policy of zero importation of solid waste by 2020 [102]. 

Meanwhile, China also introduced the standard waste classification system, which requires residents to separate waste into four categories: green for food waste, blue for recyclable waste, red for hazardous waste, and black for the refuse MSW [102]. However, much waste is still disposed of without separation by mixing with toxic and harmful substances [103]. To effectively encourage citizens to participate in waste segregation, China has introduced a bonus point incentive system for those who correctly separated waste in some cities such as Beijing, Shanghai, Hangzhou, and Xiamen [104]. At the same time, the government of China set a target for domestic recycling of at least 35% by 2021 [105]. In 2020, the treatment of MSW in China relied heavily on landfill (61%) and incineration (35%), and the rest was recycling [106]. China has 69 incinerators, 324 controlled landfills, and 20 sanitary landfills [1]. In 2018, the country’s waste incineration capacity reached 102 million tons per year (48%) and generated electricity for about 3815 kWh in 2017 [105].

### 7.4. India

Urban Local Bodies (ULBs) in India are responsible for MSW management, mainly using their own budget, human resources, and equipment [107]. The roles include but are not limited to collection, resource recovery, storage, and transportation services. India has regulated the obligation of waste generators to separate MSW into three categories: wet, dry, and hazardous wastes [108], and introduced two colored bins, the blue bin for dry waste and the green one for wet waste [109]. Waste generators have to pay the collection fee, but it is unclear how this is calculated [109].

India has developed many rules to reduce waste and environmental pollution since the 19th century, such as the Hazardous Waste and Chemical Rule (1989); the Biomedical Waste (Management and Handling) Rules (1998); the Recycled Plastic Manufacture and Use Rules (1999); the Municipal Solid Waste Rules (2000); the Battery Rules (2001); and the Electronic Waste Rules (2011) [110]. However, similar to Cambodia and other developing nations, the rules have not been successfully implemented due to the lack of public participation, limitation of public awareness and education, insufficient transportation and equipment, financial constraints, less priority on waste management, lack of technically skilled staff, and poor land acquisition for installing treatment facilities and landfill sites [107]. It is estimated that about 70% of the total MSW in Indian cities and states is collected [11,111]. To improve the MSW management in cities, the central Indian government has allocated funds for supplementing the resources to the ULBs under flagship projects since 2005, while the Ministry of Environment, Forest, and Climate Change has supported up to 50% of the capital cost for setting up pilot composting plants [108]. Currently, there are 79 composting plants nationwide, absorbing about 6% of collected waste [112]. 

India has numerous waste treatment methods, such as composting, bio-digestion, recycling, incineration, pyrolysis, waste to wealth, and WTE [113]. The conventional WTE technology has been used in India since 2003. However, it is not practical due to the high proportion of organic waste, high moisture content, high inert content, and low calorific value content [111]. Therefore, the Ministry of New and Renewable Energy (MNRD) has recently promoted the WTE projects by providing subsidies between USD 0.26M (Rs. 2 crore) and USD 1.32M (Rs. 10 crore) for the capital subsidy [108]. Currently, five WTE piloting projects have obtained this subsidy from the government [108]. Moreover, the MNRD has also announced a higher purchase rate of electricity generated from the WTE plants [108].

### 7.5. Indonesia

Indonesia generates the highest amount of MSW among Southeast Asia countries with 64 million tons per year, followed by Thailand with 26.77 million tons per year [30,114]. The waste management system in most cities in Indonesia still relies on the single collection, transportation, and landfilling method [115,116]. The collection service is provided by the private and public sectors (ULBs). Household waste is collected by the neighborhood and community organization and transported to Temporary Disposal Sites (TPS) or Intermediate Transfer Facilities (TPST), while the commercial and industrial waste is collected and transported to TPS or directly to landfill by estate owners [86]. The ULBs are responsible for collecting and transporting waste from the public and waste at the TPS/TPST to the landfills. The Indonesian government has developed numerous policies, programs, strategies and projects for MSW, but these are fully implemented and enforced at all governmental levels [30]. Source segregation is not practically implemented, as people still dispose of mixed and unsorted waste [30,116]. Waste reduction, reuse, and recycling rely on community-based solid waste management (CBSWM). 

It should be noted that CBSWM is a community-based approach involving community participation in MSW management and recycling [117]. Community members are encouraged to separate waste at household and collection sites [118]. The waste bank is established in the community where recyclable waste that is collected by residents is weighed and valued based on the type and cluster of the waste [119]. The organic waste is processed with compost technology, while the recyclable waste is reused or sold [120]. However, waste banks in Indonesia are not widely implemented [121]. To tackle the issues that arose by MSW, the Indonesian government enacted a decree to support WTE projects’ 12 biggest cities. In this sense, the national electric company has an obligation to buy electricity that is produced from WTE plants at a price of up to 14.54 cents/kWh [122]. 

### 7.6. Thailand

In Thailand, the role of MSW collection and disposal is taken up by local administration organizations (LAOs). LAOs charge the MSW collection fee from residents to cover the collection and disposal expenses [121]. Residents must separate waste at the generation source [114]. However, the level of participation is relatively low [30]. Like Cambodia, landfilling in Thailand is the most popular disposal method, followed by incineration and composting [121,123]. The efficiency of waste collection accounted for about 80% of MSW generation countrywide in 2016. Nevertheless, only 35% of the total generated waste is correctly disposed of through landfilling, composting, incinerating, and other treatment methods [84]. Recycling accounted for about 21% of total generation, slightly lower than the USA (Table 5). A large amount of waste (42%) is incorrectly disposed of via open burning or illegal burning [84,123].

Recognizing the problems of MSW, Thailand’s government has released numerous policies and campaigns to support the waste management system. The 3Rs campaign is implemented to increase waste recycling via waste banks. Waste banks in Thailand have been implemented in schools and communities, but school waste banks are largely implemented over community waste banks. There are about 500 waste banks in 30 provinces, which contribute to waste reduction by 18,000–30,000 tons per year [121]. Local communities play a crucial role in improving their MSW management, but there has recently been no incentive to support local communities in waste separation and recycling [121]. The government supports private investments in waste management through public–private partnership schemes. However, the participation of the private sector in MSW management service is still limited due to constraints in understanding the business by the financial institutions [30]. The Thailand government supports the WTE plant by setting a target for energy recovery from waste by 500 MW in 2036 [121]. Currently, there is one WTE plant with an installed capacity of 66 MW in Thailand [121]. 

### 7.7. Vietnam

The overall management of MSW in Vietnam falls under the responsibility of Provincial People’s Committees (PPCs) in the provinces or cities. The Department of Natural Resources and Environment (DONRE) is in charge of MSW management, monitoring environmental quality, and implementing the governmental policies and regulations [5]. The state-owned company, Urban Environment Company (URENCO), mainly undertakes the waste collection, transportation, treatment, management and operation of g the landfill sites. However, MSW collection in some cities is served by private companies that are in partnership with the government [5,124]. In 2020, Vietnam proposed the pay-as-you-throw model by adopting the experience of other countries such as Japan and Korea, in which the waste collection fee is charged based on the amount of waste bags needed [125]. The government gains revenue from selling plastic bags for waste packing (eco-plastic) to cover waste collection, transportation, and treatment services. By adopting this service fee model, discarded waste is expected to reduce as people would likely change their habits by sorting out waste to save money from buying waste bags. 

Vietnam has also allocated funds for MSW management, but these are limited and unequally shared among provinces and cities. More than 90% of the fund serves for waste collection and transportation, and less attention is paid to waste treatment and landfill management [76]. The present collected fee is not sufficient for operation of the service that requires a government subsidy. For example, in Ho Chi Minh city, the government subsidizes 30% on a collection fee for household waste, since the household fee charge is very low, ranging from USD 0.3 to 0.7 per month [126].

The law that obligated sorting out garbage at the source was enacted in 2010. However, the ground implementation is limited [125]. Source segregation accounts for about 8–12% of total waste generation [124]. Regarding waste collection rate, Vietnam has an average of 85% for urban areas and 40% for rural areas [5], and the highest collection rate is found in Hanoi and Ho Chi Minh at 98% and 97%, respectively [1,5,124]. Like in Cambodia, a large proportion of MSW has been sent to landfills, and a small amount has been treated at recycling facilities, composting plants, and incinerators. Vietnam has developed incentive policies, including the provision of land, technology development, capacity building, and investment loan, and by 2015, Vietnam had 105 treatment facilities: 44 incinerators, 25 composting plants, and 36 other facilities [124]. A WTE plant has been operating in Vietnam since 2016, with an installed capacity of 75 tons of industrial waste and hazardous waste inputs and an energy output of 20 MW [124].

## 8. Challenges and Priorities

As specified above, MSW management in many cities and districts of Cambodia have been improved in the last decade. However, these efforts may not be enough to address the issues that are encountered. 

The current waste collection service that is provided by private operators is still limited to only the populated and high-economic income cities where residents are capable of paying for waste collection fees. However, considerable dissatisfaction with the quality of services has been voiced, and some residents are unwilling to pay the fee, resulting in many private operators being unable to recover the operational costs and finding difficulty expanding service coverage [17,127]. Most rural areas still do not have access to the service. Hence, burying, burning, and illegally dumping on vacant land, road, and water bodies are the most practical options for residents outside the service areas [7,13,54,60], which are potentially harmful to the environment. 

The waste collection fee is another bottleneck for MSW management. The fee is calculated based on the type of house for household waste and the business size for commercial waste and varies from place to place. There is no standardization on this calculation and no consultation with local people. 

The acknowledgement of stakeholders and their interests by involving them in all processes of MSW management, ranging from planning to implementation, is a crucial success [128]. However, coordination among stakeholders involving MSW management is rather tricky, particularly at the government site. Some provincial authorities undertake operational roles instead of supporting, coordinating, and encouraging city and district authorities to manage the system under their jurisdiction [54]. 

The 3Rs are the most promising way of effectively using resources and expanding the landfill lifespan [129]. Such a good mechanism cannot be achieved without public participation, private sector involvement, and government incentive policy. The government has utilized the 3Rs mechanism since 2008, but its implementation has been rather limited due to lack of awareness [130], little cooperation from the private sector, and weak law enforcement. 

Lack of infrastructure and technology is a burning topic for MSW management in Cambodia. Inadequate vehicles and unstandardized storage systems could also be factors affecting collection efficiency. Apart from in some large cities, most vehicles are open trucks and even bullock carts, which are manually loaded and transported to the landfills without covers. Plastic bags and light waste have been seen flying from the trucks during transportation [38]. The trucks and containers are also limited and often old and unsealed. Liquid leaks out during transportation and handling activities, causing environmental pollution, generating odor, and affecting travelers and residents who live along the road. Using compactor trucks for waste transportation may reduce flying waste and the spillage of wastewater during transportation because it is specially designed and equipped with a rear compactor to lessen the waste volume, including a wastewater sump tank to collect and store leachate that is discharged from waste. However, solving these issues requires injecting more funds, which is difficult for the existing businesses. The lack of government incentive policies could be the main barrier to getting private sectors to upgrade the system and get involved in the new waste management-related businesses. 

As mentioned above, the country’s waste generation and composition data are hard to come by, and the research on the topic is minimal. The development of MSW management systems and treatment facilities requires a series of concrete and reliable data on the quantity, source, and characteristics of MSW. 

It was observed that a large amount of MSW was sent to landfills without intermediate treatments due to financial challenges and an absence of large-scale recycling facilities and treatment plants. Therefore, there is still room for waste recycling and treatment investments, but this may require some incentive supports from the government. Furthermore, an investment in waste treatment in individual cities may not be profitable for a large-scale project because of the small volume of collected waste. Nevertheless, there is no centralized MSW treatment system in place. 

Landfill is another concern for Cambodia as most existing landfills are open dumping and located in low-lying areas, possessing a high risk of environmental pollution and water contamination [10]. A lack of sanitary landfills may have a fatal effect on human health and environmental pollution. 

Addressing the above challenges and achieving sustainable MSW management would consume both time and tremendous efforts from the public sector (national and sub-national levels), private sector, development partners, and residents. In this regard, some key priorities should be considered for dealing with current and future waste management issues:

(1) *Competitive service provision.* To avoid the environmental burden from uncollected waste and to increase the efficiency of the MSW collection service, the competitive tendering with a clear operational plan should be transparently performed, and the short-term contract adopted. Evidence of the deficiency of service provision was seen in Phnom Penh, where the service was monopolized for a long-term contract (49 years) [38];
(2) *Redefine service fee*. Willingness to pay for the service is key to a successful MSW management system. A standard service fee setting model should be advised, notwithstanding different urban areas, and the successful experience from other countries should not be overlooked. i.e., “pay-as-you-throw” adopted in Japan, Korea, and Vietnam, or waste/sanitation tax in Switzerland and India;
(3) *Stakeholder engagement*. Sustainable waste management cannot be achieved without involvement from all stakeholders, including the national government in developing policy and strategy frameworks, sub-national authorities in implementing the policy and management of the system, the private sector in providing the service, development partners in conducting research and development and providing financial support, and residents in waste separation;
(4) *Capacity building and awareness-raising*. The participation of local people is a driving force in achieving the 3Rs strategy by reducing waste disposal and sorting out waste for recycling. Hence, environmental education is vital for both formal and informal education systems. Informal education can be made through environmental campaigns, media, and social media, while formal education can be made by integrating environmental education into the school curriculum. An incentive program should also be introduced, for example, the bonus point incentive system, implemented in some cities of China;
(5) *Promoting resource recovery*. In a circular economy, waste is regarded as a resource. The private sector and local people get involved by reducing consumption and waste generation, reusing materials, recycling (recyclable and compostable), and recovering energy from the remaining materials before they up in landfill. The existing informal recyclers should be promoted and upgraded. WTE incineration has also been seen as an alternative to landfill. It diverts the waste that remains after recycling into energy or steam, reducing the environmental burden at the landfills and minimizing the investment in landfill construction. However, this system is highly technological and requires a huge investment cost, which requires a subsidy from the government to secure its sustainability;
(6) *Community-based solid waste management formation*. Cambodia already has many forms of community-based organizations. Therefore, it is important to learn about the experience of Thailand and Indonesia in improving waste recycling and management via waste banks. The potential application of waste banks should be in schools, pagodas, communities, or a combination thereof;
(7) *Formulation of incentive provision.* An enabling environment should be created for the MSW management sector by regulating incentive-supporting policy and strategy. Several incentive mechanisms include capital cost subsidy, import tax exemption, tax breaks, material bans, and material controls [1];
(8) *Application of advanced digital technologies in MSW management.* The use of digital technology offers a new generation approach to improving waste management systems effectively. Artificial Intelligence (AI) has been seen as a powerful technology increasingly attaining popularity and application in many sectors, including MSW management [131]. The emerging technologies have been applied in many areas, including waste generation, bin level monitoring (Internet of Things), waste collection and vehicle route, waste sorting, waste treatment, and waste management planning [132]. Incorporating AI in MSW infrastructure has been successful in many developed countries in Europe, Asia, and America to increase the recycling rate, minimize labor, reduce cost, maximize efficiency, and improve management methods [131,132]. Hence, shifting conventional MSW management methods to AI-based systems should be an option for Cambodia to foster its circular economy strategy in the industry 4.0 era.
(9) *Enhancement of data collection*. The unavailability of data is the critical barrier to developing management systems, designing treatment facilities, and financial resources allocation. Hence, to help the government and private sector develop a strong management plan, great efforts should be made to collect such extensive data and make them publicly available. The research and development on this topic should also be accelerated.

## 9. Conclusions

This review paper gives a comprehensive update on the situation and management of MSW in Cambodia and the efforts of the government to overcome the MSW management issues, which are concomitantly compared with other developing and developed countries. The review highlights the limitations of the aspects of the MSW management system and technologies and suggests possible actions for sector improvement. Sustainable MSW management in Cambodia requires strong participation from the public, private sector, development partners, and government. The service provision is only available in urban areas with a 72% collection efficiency. Most uncollected wastes are burned and discharged into land and water bodies, causing severe environmental pollution. Service providers should quickly expand and efficiently improve their managing and treating capacities in MSW terms. The government incentive policy is indispensable for improving the existing systems and extending resource recovery to attain the country’s circular economy. Therefore, extensive novel studies should be carried out to investigate the existing MSW management systems and their impacts on the environment. Moreover, advanced technologies and synchronous management systems such as Artificial Intelligence (AI) and the Internet of Things (IoT) should be appropriately applied to increase management efficiency and meet sustainable development goals.

## Figures and Tables

**Table 1:** Estimation of MSW generation based on population for 2008–2020.

Year	Population (million) ^a^	GDP ^a^			MSW Generation (million ton)		Per Capita ^c^ (kg/day)
		Per Capita (USD)	Annual (million USD)	Annual Growth (%)	^b^	^c^	
2008	13.88	746	10,352	6.69	3.74	3.71	0.73
2009	14.09	738	10,402	0.09	3.78	3.79	0.74
2010	14.31	786	11,242	5.96	3.85	3.85	0.74
2011	14.54	882	12,830	7.07	3.91	3.92	0.74
2012	14.78	951	14,054	7.31	3.99	3.99	0.74
2013	15.03	1013	15,228	7.36	4.09	4.06	0.74
2014	15.28	1093	16,703	7.14	4.16	4.14	0.74
2015	15.52	1163	18,050	7.12	4.18	4.41	0.78
2016	15.77	1270	20,017	6.94	4.24	4.49	0.78
2017	16.01	1385	22,177	6.84	-	4.58	0.78
2018	16.25	1512	24,572	7.47	-	4.67	0.79
2019	16.49	1643	27,089	7.05	-	4.69	0.78
2020	16.72	1513	25,291	−3.14	-	4.78	0.78

Source: Data from ^a^ [26], ^b^ [24], ^c^ [27].

**Table 2:** Comparison of waste composition among provinces.

Province	Waste Composition (%)							
	Food Waste	Paper	Plastic	Metals	Textile	Glass	Wood and Dry Matter	Other
Country ^a^	55	3	10	7	-	8	-	17
Phnom Penh ^b^	49	7	21	1	8	1	7	6
Battambang ^c^	71	2	10	3	2	4	6	2
Siem Reap ^c^	54	6	11	1	3	3	11	11
Kampong Cham ^c^	60	5	12	1	1	2	3	16
Kampong Chhnang ^d^	80	2	3	8	1	1	-	-
Pursat ^e^	50–65	2–4	10–15	2–6	2–4	4–6	1–2	10–15
Kampong Thom ^f^	61	5.3	13.5	0.6	3.7	2.6	3	4

Source: Data from ^a^ [32], ^b^ [33], ^c^ [34], ^d^ [23], ^e^ [35], ^f^ [36].

**Table 3:** Waste collection in the city and district centers of Cambodia in 2021.

No	Province/ Municipality	Cities/ Districts (number)	Cities/ Districts with Service (number)	MSW Generation (ton/day)	MSW Collection (ton/day)	Collection Efficiency (%)	Landfill (number)	Landfill Size (ha)	Incinerator (number)
1	Banteay Meanchey	9	9	310	228	74	11	45.50	2
2	Battambang	14	11	365	234	64	14	13.20	3
3	Kampong Cham	10	10	173	130	75	14	15.50	1
4	Kampong Chhnang	8	7	125	41	33	5	23.90	-
5	Kampong Speu	8	8	91	68	75	12	31.10	4
6	Kampong Thom	9	8	146	49	34	10	75.00	3
7	Kampot	9	8	98	77	79	8	13.10	-
8	Kandal	11	11	561	518	92	10	31.90	5
9	Koh Kong	7	4	106	106	100	2	4.30	1
10	Kratie	6	4	101	40	40	6	44.00	1
11	Mondul Kiri	5	4	20	11	55	4	25.50	2
12	Phnom Penh	14	14	3076	2830	92	1	31.00	6
13	Preah Vihear	8	3	63	31	49	5	28.50	3
14	Prey Veng	13	12	767	123	16	9	10.00	2
15	Pursat	7	7	331	301	91	11	7.50	2
16	Ratanak Kiri	9	4	87	20	23	6	16.50	-
17	Siem Reap	12	11	316	234	74	11	25.60	4
18	Preah Sihanouk	5	5	285	273	96	2	100.50	6
19	Stung Treng	6	1	65	22	34	2	101.00	-
20	Svay Rieng	8	8	381	224	59	4	4.50	1
21	Takeo	10	10	166	32	19	7	4.90	4
22	Oddar Meanchey	5	5	106	41	39	4	19.70	1
23	Kep	2	2	78	50	64	1	13.39	1
24	Pailin	2	2	53	19	36	1	5.50	-
25	Thboung Khmum	7	7	93	47	51	4	9.50	2
	**Total**	**204**	**175**	**7963**	**5749**	**72**	**164**	**701.09**	**54**

Source: Data from [18,27].

**Table 4:** Recyclable waste purchased by junkshops from 2010 to 2021.

Year	Junkshop (number) ^a^	Recyclable Waste (ton) ^a^						Revenue Recovered (USD) ^b^
		Paper and Cardboard	Plastic	Aluminum	Iron	Glass	Total	
2010	259	26,522	23,583	8449	63,077	1367	122,997	25,578,460
2011	313	25,340	21,689	7297	68,336	14,469	137,130	25,415,510
2012	421	14,856	13,960	9866	30,013	9083	77,777	19,556,430
2013	450	18,004	12,820	10,113	19,955	13,652	74,544	18,559,020
2014	483	22,838	15,842	15,942	29,930	14,311	98,863	27,426,040
2015	439	14,769	12,678	100,050	17,184	9028	153,709	114,686,375
2016	445	33,857	30,907	87,741	74,328	20,134	246,967	115,820,995
2017	462	63,719	28,871	89,430	105,770	16,256	304,046	124,993,065
2018	462	36,829	23,886	26,664	45,723	7269	140,371	43,796,765
2019	498	38,180	27,208	27,973	46,282	7532	147,175	45,972,940
2020	NA	NA	NA	NA	NA	NA	NA	-
2021	692	67,701	36,389	31,120	64,348	2170	201,728	56,267,615

Source: Data from ^a^ [27], ^b^ authors’ own calculation based on [33], NA: Data not available.

**Table 5:** Comparison of MSW management with other countries.

Parameters	USA	Japan	China	India	Cambodia	Indonesia	Thailand	Vietnam
Population (million)	331.5	125.8	1410.0	1380.0	16.7	254.5	70.1	97.3
MSW generation (million ton)	262.0	42.7	428.1	52.0	4.8	64	27.1	27.8
MSW generation rate (kg/capita/day)	2.03	0.93	0.73	0.85	0.78	0.70	1.14	0.80
MSW management role	States	Local government	Local government	Local government	Local authorities	Local authorities	ULBs	DONRE
MSW service provider	Local government and private	Local government and private	Local authorities	ULBs	Private	ULBs and private	LAOs	URENCO
Source segregation rule	Yes	Yes	Yes	Yes	No	Yes	Yes	Yes
MSW collection model	Door to door	Collection point	Community bins	Door to door	Door to door	TPS	Curbside	Door to door
MSW collection rate (%)	100	100	98	70	56	45–50	80	85
Landfilling (%)	53	1	61	75	44	66.4	29	63
Recycling (%)	26	19	4.4	10–20	4	5	21	10
Composting (%)	8.9	-	3	-	2	7	2	-
Incineration (%)	13	80	35	14	4	2	3	14
WTE plants (number)	86	358	69	11	0	-	1	10
Incentive policy	Yes	Yes	Yes	Yes	No	Yes	No	Yes

Sources: Data from [1,79,80,81,82,83,84,85,86,87,88,89,90,91,92,93,94]. DONRE, Department of Natural Resource and Environment; ULBs, Urban Local Bodies; URENCO, Urban Environmental Company; LAOs, Local Administration Organizations; TPS, Temporary Disposal Site.
